# In situ atomistic observation of disconnection-mediated grain boundary migration

**DOI:** 10.1038/s41467-018-08031-x

**Published:** 2019-01-11

**Authors:** Qi Zhu, Guang Cao, Jiangwei Wang, Chuang Deng, Jixue Li, Ze Zhang, Scott X. Mao

**Affiliations:** 1 Center of Electron Microscopy and State Key Laboratory of Silicon Materials, School of Materials Science and Engineering, Zhejiang University, Hangzhou, 310027 China; 2 Department of Mechanical Engineering, University of Manitoba, Winnipeg, MB R3T 5V6 Canada; 3 Department of Mechanical Engineering and Materials Science, University of Pittsburgh, Pittsburgh, PA 15261 USA

## Abstract

Shear-coupled grain boundary (GB) migration is of general significance in the deformation of nanocrystalline and polycrystalline materials, but comprehensive understanding of the migration mechanism at the atomic scale remains largely lacking. Here, we systematically investigate the atomistic migration of Σ11(113) coherent GBs in gold bicrystals using a state-of-art in situ shear testing technique combined with molecular dynamic simulations. We show that shear-coupled GB migration can be realised by the lateral motion of layer-by-layer nucleated GB disconnections, where both single-layer and double-layer disconnections have important contributions to the GB migration through their frequent composition and decomposition. We further demonstrate that the disconnection-mediated GB migration is fully reversible in shear loading cycles. Such disconnection-mediated GB migration should represent a general deformation phenomenon in GBs with different structures in polycrystalline and nanocrystalline materials, where the triple junctions can act as effective nucleation sites of GB disconnections.

## Abstract (web summary)

Shear-induced grain boundary migration at the atomic level is still not well understood. Here the authors combine in situ shear testing experiments and molecular dynamic simulations to reveal the atomistic mechanism of disconnection-mediated GB migration in different gold nanostructures.

## Introduction

Grain boundary (GB) migration is a prevalent plastic deformation mode in nanocrystalline^1–4^ and polycrystalline^5,6^ materials, and a comprehensive understanding of GB migration is vital to the development of novel materials through GB engineering. Numerous theoretical and experimental investigations have been conducted to understand the underlying GB migration mechanisms^7–10^, and several shear-coupled GB migration models have been proposed correspondingly, including the displacement shift complete (DSC) dislocation model^11,12^, Cahn model^13,14^, pure shuffling model^15,16^ and shear migration geometrical model^17,18^. The DSC model^11,12^ suggests that GB migration is a result of the lateral motion of DSC dislocations (also termed as disconnections) on the boundary plane. The Cahn model^13,14^, extended from Read and Shockley’s pioneer description of low-angle GB motion^19^, predicts < 100 > and < 110 > branches of coupling for [001] tilt GBs according to the slip directions of GB dislocations. The pure shuffling model^15,16^ proposes that the migration of high angle GB is mainly mediated by the local conservative shuffling of atoms at pure steps or ledges with no shear displacement. Recently, the shear migration geometrical model^17,18^ has been introduced to account for the low coupling factors in experiments and is thus more applicable to quantify the GB-based plasticity in real polycrystals.

Some experimental insights into the GB migration have been obtained in the past two decades, with the spring up of well-designed in situ transmission electron microscopy (TEM) techniques^10,20,21^. For example, in situ heating experiments have revealed a thermal-induced GB migration in Au and Al thin films at 0.4–0.7 *T*_m_ (*T*_m_ is the melting temperature) through the atomic shuffling^22^ or GB disconnection^23^ mechanisms. Numerous in situ straining of nanocrystalline and polycrystalline materials^6,10,24,25^ have also been conducted, however only microscale GB migration and dislocation/step motion were observed. Currently, the atomistic mechanism of disconnection dynamics (including the nucleation, propagation and interaction) and their contribution to the GB deformation remain largely unclear, especially in experiments due to the technical limitations.

Here we use the Au bicrystal nanojunctions with the Σ11(113) coherent GB as an example to investigate the atomistic mechanism of shear-induced GB migration. Σ11(113) coherent GB is a representative low energy < 110 > tilt GB in bulk face-centred cubic (FCC) metals, which possesses an GB energy only higher than that of Σ3 coherent twin boundary^26^ and thus has been frequently investigated in both experiments^7,23^ and simulations^27,28^. Although a disconnection-mediated migration of Σ11(113) GB has been proposed^23^, the underlying atomistic mechanism remains largely unclear. By conducting the state-of-art in situ shear testing and molecular dynamic (MD) simulation, we revealed the atomistic migration mechanism of the Σ11(113) coherent GB coupled to the lateral motion of layer-by-layer nucleated GB disconnections with the height of either one or two atomic layers. These two types of disconnections can transform to each other dynamically via the frequently-occurred composition and decomposition processes, contributing to a fully-reversible GB migration in shear loading cycles. We further demonstrate that the disconnection-mediated GB migration is a general deformation phenomenon in GBs with different structures and the triple junctions can serve as effective nucleation sites of GB disconnections, enriching our understanding of GB-mediated plasticity in polycrystalline and nanocrystalline materials.

## Results

### Disconnection-mediated migration of Σ11(113) coherent GB

**Fig. 1:**
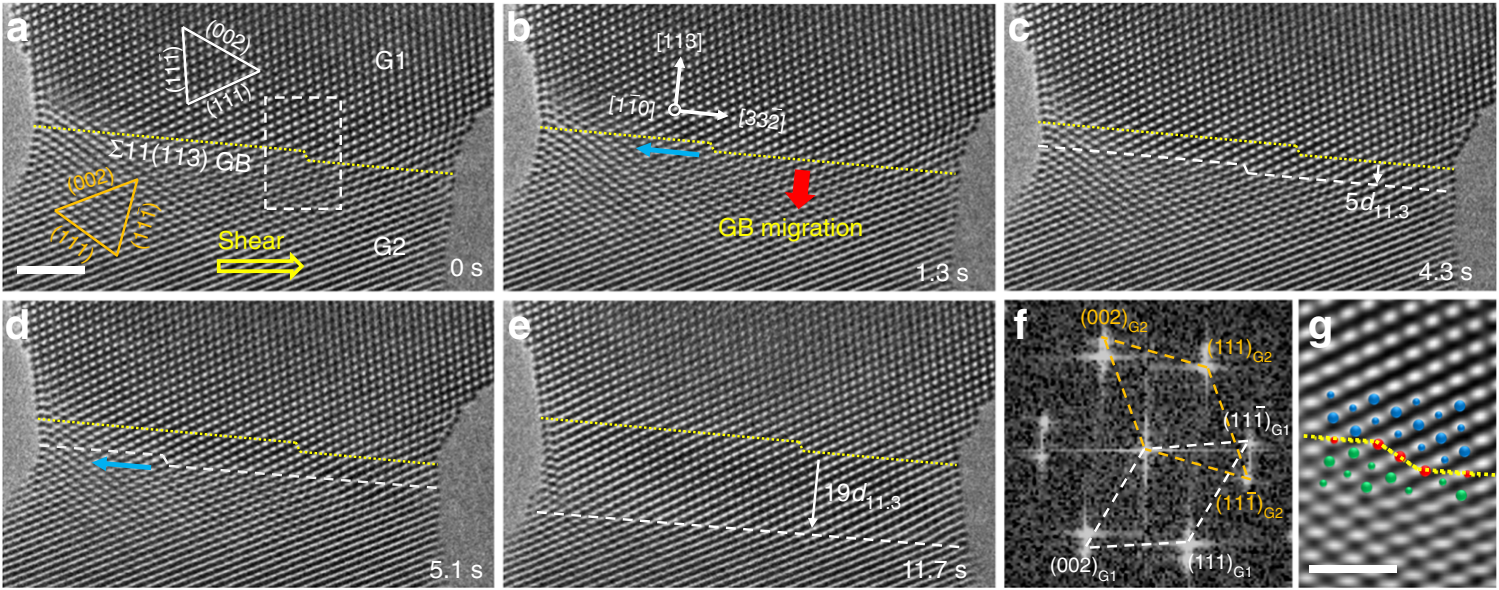
Shear-induced migration of a Σ11(113) coherent grain boundary (GB) in an Au bicrystal. **a** Structure of an as-fabricated Au bicrystal with a Σ11(113) coherent GB. The lattices of the upper and bottom grains in this bicrystal showed a symmetrical relation. Close observation shows that a disconnection pre-existed on the GB. A shear stress was then applied to the bottom grain (G2) with an inclination of ~5° to the GB plane, as indicated with the yellow arrow. **b** GB migration mediated by the lateral motion of GB disconnection along the direction of the upper grain. The directions of GB migration and disconnection motion are indicated by the red and blue arrows, respectively. **c**, **d** Sequential deformation snapshots showing the disconnection-coupled GB migration under shear loading. **e** Successive migration of the GB with a total distance of 19*d*_113_. The yellow dotted lines and white dashed lines in (**c**–**e**) represent the initial and current positions of the GB, respectively. **f** Fast Fourier transform pattern confirms the coherent relation across the GB in (**a**). **g** Filtered high resolution image showing the detailed structure of the disconnection core. The schematic superimposed on (**g**) presents the atomic structure of the GB disconnection. Large and small particles represent the atoms located on the neighbouring planes. Scale bars: (**a**) 2 nm, (**g**) 1 nm

Before deformation, an Au bicrystal nanojunction with a Σ11(113) coherent GB was fabricated by the in situ nanowelding inside TEM (see Methods). Atomistic observation in Fig. 1a indicates that the lattices in the two grains of Au bicrystal show a symmetrical relation, as demonstrated by the fast Fourier transform pattern in Fig. 1f. Due to the orientation difference, a Σ11(113) coherent GB (indicated by the yellow dotted line in Fig. 1a) was formed between these two grains. High resolution TEM image and the superimposed schematic in Fig. 1g show that an atomistic step with the height of two (113) lattice spacings pre-existed on this Σ11(113) coherent GB, which was usually denoted as GB disconnection in literature^24,27,29^. These structural features of the Σ11(113) GB match well with the ones reported in MD simulation^28^ and bulk bicrystal^23^. It is also noticed that neither lattice dislocation nor GB dislocation can be identified in the as-fabricated Au bicrystals (Supplementary Fig. 1). Subsequently, a shear loading was applied to the bottom grain (denoted as G2) of the bicrystal at a constant rate (~0.005 nm s^−1^), and the loading direction (indicated by the yellow arrow in Fig. 1a) was close to the GB plane with an inclination of ~5°.

Upon deformation, it is interestingly observed that no lattice defect was emitted from the Σ11(113) coherent GB or the pre-existed GB disconnection (Supplementary Movie 1), in contrast to the widely-observed GB nucleation of partial dislocation and twin in the deformation of nanocrystalline materials^30–34^. Instead, continuous and smooth migration of the Σ11(113) coherent GB occurred, which proceeded through the lateral motion of GB disconnections, as demonstrated by the deformation snapshots in Fig. 1b–e and Supplementary Movie 1. Under shear loading, the pre-existed GB disconnection moved leftward gradually along the $\left[ {\overline 3 \overline 3 2} \right]$ direction of the upper grain with an average speed of 2.22 nm s^−1^ (indicated by the blue arrow in Fig. 1b), which eventually annihilated at the free surface. Associated with the lateral motion of this GB disconnection was a downward migration of the Σ11(113) coherent GB (shown by the red arrow in Fig. 1b). Afterwards, a new GB disconnection was emitted from the free surface under the shear loading (the surface nucleation of GB disconnections cannot be clearly identified due to the ultrafast process and the localised rearrangement of atoms on the surface), which contributed to the subsequent migration (Fig. 1c, d). With the sequentially-nucleated GB disconnections, successive GB migration occurred and proceeded in the same manner via the disconnection propagation and annihilation (Fig. 1e). In five individual samples with Σ11(113) coherent GB, similar disconnection-mediated GB migration phenomena were captured during the in situ shear testing. Supplementary Fig. 2 presents an additional example of such disconnection-mediated migration of Σ11(113) coherent GB.

### Atomistic mechanism of GB disconnection motion

**Fig. 2:**
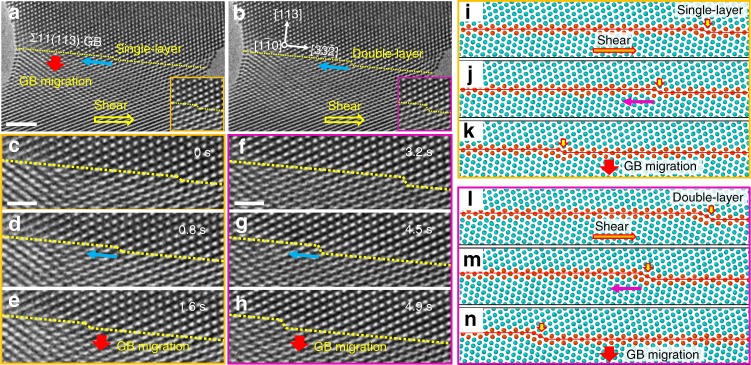
Atomistic mechanism of GB migration through the lateral motion of single-layer and double-layer disconnections. **a**, **b** Typical single-layer (**a**) and double-layer (**b**) disconnections observed during the migration of Σ11(113) GB. The directions of shear stress, disconnection motion and GB migration are indicated with the yellow, blue and red arrows, respectively. Insets in (**a**, **b**) show the core structures of corresponding disconnections. **c**–**h** Deformation snapshots further reveal the atomistic mechanism of GB migration due to the lateral motion of single-layer (**c**–**e**) and double-layer (**f**–**h**) disconnections, respectively. The direction of disconnection motion was opposite to that of the shear stress, with the average motion rates of single-layer and double-layer disconnections measured to be 1.96 nm s^−1^ and 2.42 nm s^−1^, respectively. **i**–**n** Molecular dynamic (MD) simulation shows lateral motion of single-layer (**i**–**k**) and double-layer (**l**–**n**) disconnections coupled to the continuous migration of the Σ11(113) coherent GB. The directions of shear stress, disconnection motion and GB migration are marked out by the orange, purple and red arrows, respectively. The simulations were performed at 300 K with a shear stress applied to the bottom grain. The disconnections are pointed out by the yellow arrows in each snapshot for clear demonstration of their continuous motion. Scale bars: (**a**) 2 nm, (**c**) and (**f**) 1 nm

As demonstrated in Fig. 1, continuous migration of the Σ11(113) coherent GB was a direct result of the lateral motion of sequentially-nucleated GB disconnections. To gain further insights into the migration mechanism, atomistic deformation snapshots were further analysed by focusing on the disconnection dynamics. Frame-by-frame analysis indicates that two different types of GB disconnections with the height of either one or two (113) atomic layers were frequently generated under the shear loading (Fig. 2a, b), while no lattice defect can be identified at the cores of these GB disconnections (Insets in Fig. 2a, b). During deformation, the lateral motion of both the single-layer (Fig. 2c–e) and double-layer (Fig. 2f–h) GB disconnections proceeded continuously, resulting in a downward migration of this Σ11(113) coherent GB for a distance of one and two (113) lattice spacings, respectively. In both cases, the directions of disconnection motion were opposite to those of the applied shear loading (Fig. 2d, g). It is also noticed that multiple GB disconnections can be emitted sequentially and co-existed in the bicrystal on the neighbouring (113) planes (Supplementary Fig. 3). As a consequence, the Σ11(113) coherent GB showed a layer-by-layer migration behaviour due to the successive nucleation and lateral motion of GB disconnections, similar to the deformation twinning in FCC metals^35,36^. MD simulations reveal that the single-layer and double-layer disconnections possess tiny Burgers vectors of 1/22〈471〉 and 1/22〈332〉, respectively, which is consistent with the previous MD simulation results^27,28^ and experimental measurement^7^. As demonstrated in Fig. 2i–k, l–n, the leftward motion (opposite to the shear direction) of both single-layer and double-layer disconnections were accompanied by a downward migration of the Σ11(113) coherent GB, while no emission of lattice defect from the GB was observed, consistent with our experimental observations. Atomistic displacement vector analysis in Supplementary Fig. 4 further confirms that the lateral motion of GB disconnections was merely controlled by the localised fluctuations of atoms in the disconnection core around their equilibrium positions, without the involvement of any conventional lattice dislocation.

### Composition and decomposition of GB disconnections

**Fig. 3:**
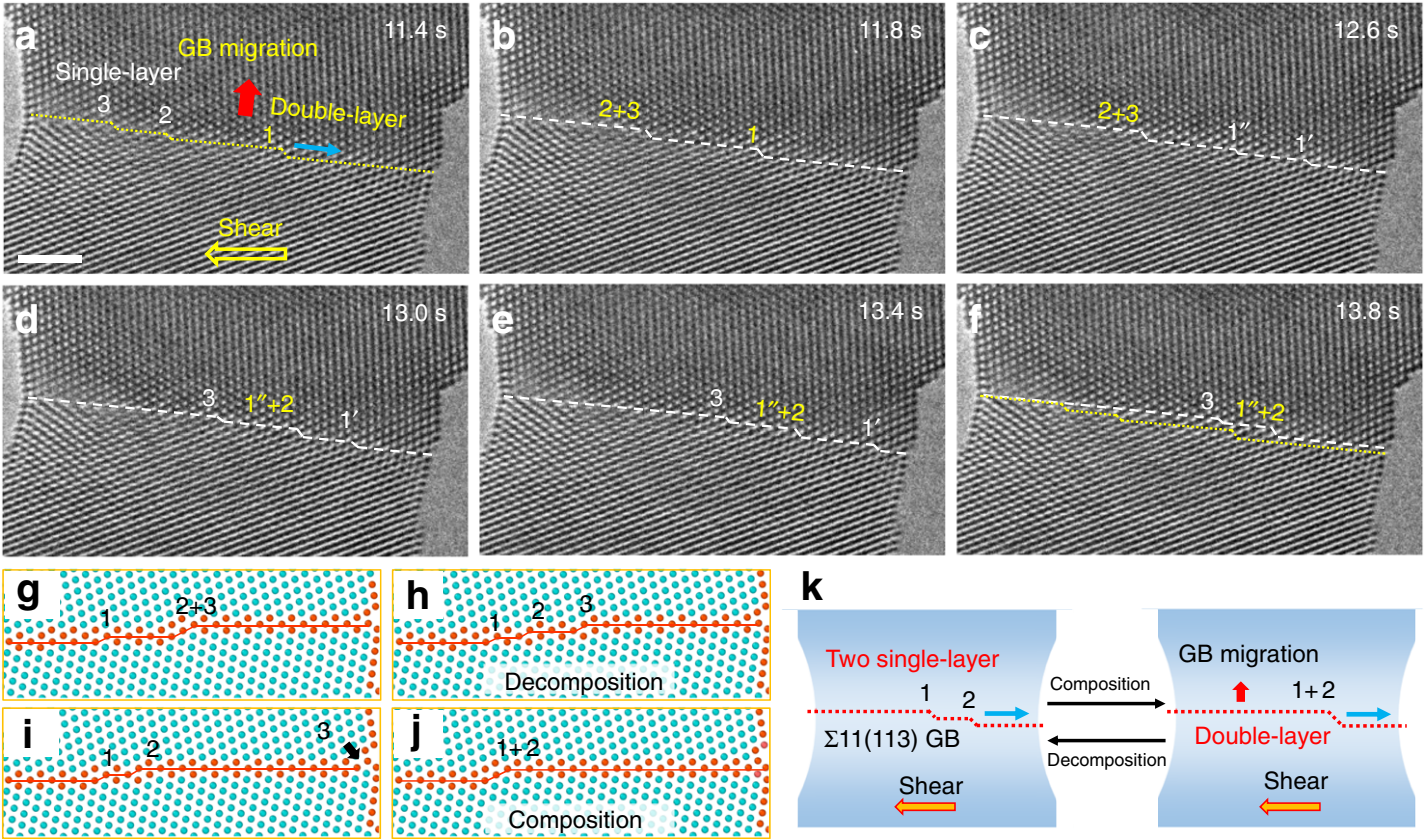
Composition and decomposition of disconnections during GB migration. **a** A Σ11(113) coherent GB with the co-existence of two single-layer disconnections (denoted as 2 and 3, respectively) and a double-layer disconnection (denoted as 1). The directions of shear stress, disconnection motion and GB migration are indicated with the yellow, blue and red arrows, respectively. **b** Composition of single-layer disconnections 2 and 3 into a double-layer disconnection (2 + 3) under shear loading, while the double-layer disconnection 1 remained almost static. **c** Decomposition of the double-layer disconnection 1 into two single-layer ones 1′ and 1″. **d** Decomposition of the double-layer disconnection (2 + 3) into two single-layer ones (2 and 3), followed by prompt composition of 2 and 1″ into a new double-layer disconnection (1″+2). **e**, **f** Lateral motion of the disconnections and the annihilation of disconnection 1′ at the free surface, resulting in the upward migration of the Σ11(113) coherent GB. The yellow dotted lines and white dashed lines represent the initial and current positions of the GB, respectively. **g**–**j** MD simulation snapshots performed at 0 K by the energy minimisation, demonstrating the dynamic processes of (**h**) decomposition of a double-layer disconnection (2 + 3), **i** subsequent surface annihilation of a single-layer disconnection 3 and (**j**) composition of the single-layer disconnections 1 and 2 into a double-layer disconnection. **k** Schematic illustrating the composition and decomposition between the single-layer and double-layer disconnections. Scale bar: 2 nm

The co-existence of different types of GB disconnections may provide a possibility for the dynamic interactions between them, given the considerable variations between their motion rates (Supplementary Fig. 5). Indeed, frequent composition and decomposition of GB disconnections were observed, which dominated the shear-coupled migration of Σ11(113) coherent GB (Fig. 3 and Supplementary Fig. 6). Figure 3a shows that different types of GB disconnections can co-exist on the Σ11(113) coherent GB, *e.g*. a double-layer disconnection (denoted as 1) and two single-layer disconnections (denoted as 2 and 3, respectively) at 11.4 s. Further shear loading caused a combination of the two single-layer disconnections (2 and 3) into a new double-layer disconnection (denoted as 2 + 3), while the original double-layer disconnection 1 remained almost static (Fig. 3b). Subsequently, the new double-layer disconnection (2 + 3) glided continuously along the $\left[ {33\overline 2 } \right]$ direction and contributed to the GB migration (Fig. 3c); in the meantime, the original double-layer disconnection 1 decomposed into two single-layer disconnections (labelled as disconnections 1′ and 1′′ in Fig. 3c, respectively). Thereafter, the double-layer disconnection (2 + 3) disassociated into two single-layer ones and its front component further composed promptly with the newly-produced single-layer disconnection 1′′ into a new double-layer disconnection (1′′ + 2), as shown in Fig. 3d. Afterwards, continuous lateral motion of these three disconnections dominated the deformation before the eventual annihilation of disconnection 1′ at the free surface (Fig. 3e, f). Associated with the dynamic composition and decomposition of these disconnections was the upward migration of the GB, as demonstrated by the distance between the initial and final positions of the GB in Fig. 3f. Fig. 3k schematically illustrates the dynamic processes of disconnection composition and decomposition.

Such dynamic interactions between GB disconnections should be governed by the total energy of the system. Supplementary Table 1 shows that the single-layer disconnections typically possess a lower surface nucleation energy (1.61 eV) than that of double-layer disconnections (3.17 eV). As a result, the surface nucleation of single-layer disconnection should be more favourable under shear loading. However, the co-existence of single-layer disconnections on the GB may be in a quasi-equilibrium state since the combination of two single-layer disconnections into a double-layer disconnection can further reduce the system energy (Supplementary Table 1). MD simulations reproduced such dynamic composition of disconnection, as shown in Fig. 3i, j. Energy minimisation performed at 0 K (without shear stress applied) shows that the composition of single-layer disconnections is a justifiable way for reducing the overall energy of the GB (Supplementary Fig. 7). In contrast, the decomposition of double-layer disconnection seems to be energetically unfavourable; however, it did occur especially in the cases when a double-layer disconnection was blocked by other disconnections, as demonstrated by the decomposition of double-layer disconnection (2 + 3) in Fig. 3g, h, which was consistent with the experimental observations in Fig. 3c, d. Such decomposition process probably originated from the repulsive force between two disconnections with the same Burgers vector, *i.e*. the approaching single-layer disconnection (**b**_1_) and the single-layer component (**b**_1_) of the double-layer disconnection (**b**_1_ + **b**_2_), which increased significantly with the decreasing distance in between. Subsequently, further composition of disconnections 1 and 2 occurred by forming a double-layer disconnection 1 + 2 (Fig. 3i, j), while the disconnection 3 escaped to the free surface (Fig. 3i). With the introduction of shear stress and finite temperature, these dynamic processes should occur more frequently. For example, MD simulation in Supplementary Fig. 8 even shows that the applied shear stress could cause the disassociation of a single-layer disconnection into a kinked disconnection dipole, which was composed of a single-layer disconnection and a double-layer disconnection. In the subsequent deformation, these two components moved oppositely and contributed to the GB migration. The dynamic propagation processes of disconnections, as well as the surface nucleation, dominated the whole shear deformation and contributed to the overall migration of the Σ11(113) GB.

### Reversible migration of Σ11(113) GB

**Fig. 4:**
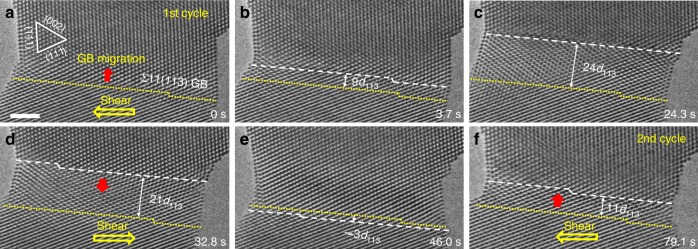
Reversible GB migration in shear loading cycle. **a**–**c** Upward migration of the Σ11(113) coherent GB under the leftward shear stress (shown by the yellow arrow) in the first loading cycle. A maximum migration distance of 24*d*_113_ was obtained before the loading was reversed. **d**, **e** Downward migration of the Σ11(113) coherent GB under the rightward shear stress. The GB migrated downward gradually with a total distance of 27*d*_113_. **f** The second shear loading cycle where the leftward shear stress induced an upward GB migration for 11*d*_113._ The yellow dotted lines and white dashed lines represent the initial and current positions of the GB in each snapshot, respectively. Scale bar: 2 nm

The disconnection-mediated plastic deformation was found completely recoverable, as manifested by the reversible GB migration in the shear loading cycles in Fig. 4 and Supplementary Movie 2. When a leftward shear stress was applied, an upward migration of the Σ11(113) coherent GB was observed through the lateral motion of GB disconnections, as demonstrated by Fig. 4a–c. However, a downward migration of this Σ11(113) GB occurred immediately once the applied shear stress was tuned to the opposite direction (*i.e*. the rightward direction, Fig. 4d, e), showing a fully reversible behaviour. Similarly, the downward migration of this Σ11(113) GB was also mediated by the lateral motion of layer-by-layer nucleated GB disconnections, proceeding in the same fashion as the upward migration but in the opposite direction. In the first loading cycle, the Σ11(113) GB migrated upward for a distance of 24*d*_113_ firstly and then reversed to move downward for a distance of 27*d*_113_ (*d*_113_ represents the lattice spacing of the (113) planes). Then, the GB migrated upward for a distance of 11*d*_113_ in the second loading cycle (Fig. 4f). Additional example in Supplementary Fig. 9 and MD simulation in Supplementary Fig. 10 further demonstrate the reversible deformability of Au bicrystals with Σ11(113) GB. More interestingly, such reversible GB migration was completely retained in further loading cycles, as long as no conventional displacive lattice defect (*e.g*. partial dislocation and nanotwin) was generated. Hence, this reversible migration of Σ11(113) GB is similar to the twinning-mediated pseudoelasticity in metallic nanowires^37,38^, which could effectively dissipate the deformation energy and benefit the cyclic deformation of nanostructured materials.

### Disconnection-mediated migration of different GBs

**Fig. 5:**
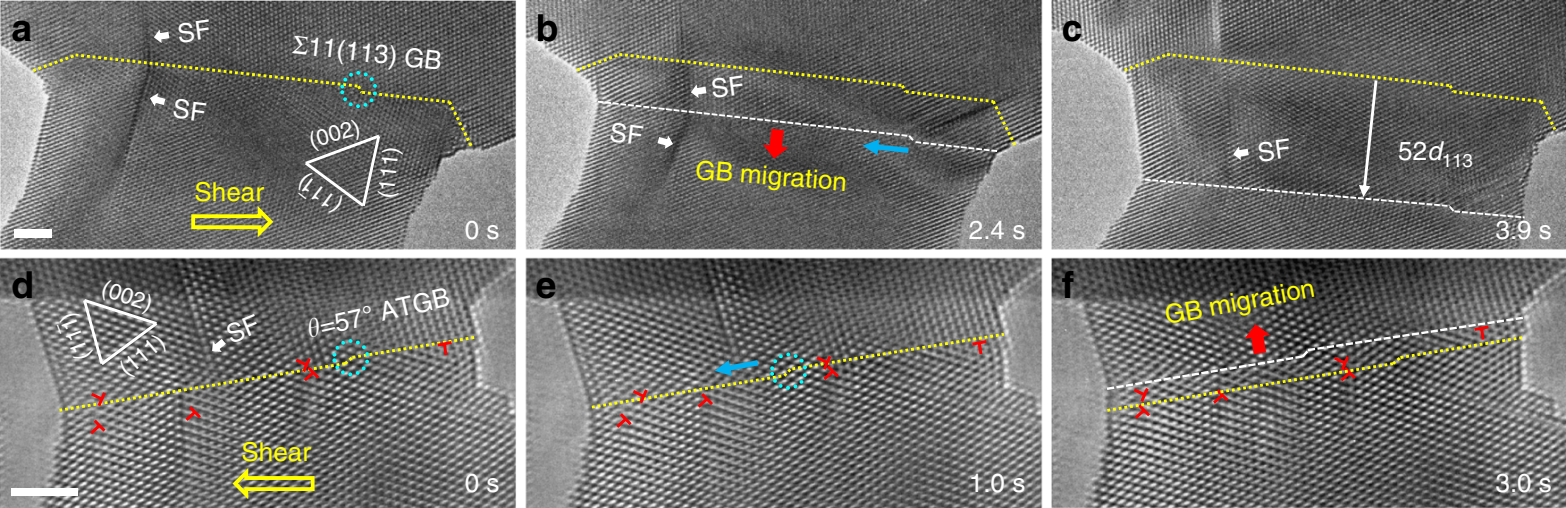
Disconnection-mediated migrations of different GBs in Au bicrystals. **a**–**c** Disconnection-mediated migration of a Σ11(113) coherent GB in an Au bicrystal with pre-existed stacking faults (SFs, indicated by the white arrows). Similarly, the successive motion and interplay of GB disconnections caused a downward migration of this Σ11(113) GB, and the presence of SFs had limited influence on the GB migration. **d**–**f** Upward migration of a *θ* = 57° < 110 > asymmetrical tilt GB (ATGB) with numerous pre-existed full dislocations near the GB. **d**, **e** The GB disconnection moved laterally towards left under shear loading and passed a pair of full dislocations, without any evident interaction. **f** Successive lateral motion of disconnections caused an upward migration of the GB. The dislocations near the GB remained almost static and had limited effect on the lateral motion of disconnections. The aqua circles in (**a**), (**d**) and (**e**) mark out the pre-existed GB disconnections; the yellow, blue and red arrows indicate the directions of shear stress, disconnection motion and GB migration, respectively. The yellow dotted lines and the white dashed lines represent the initial and final positions of the GBs, respectively. Scale bars: 2 nm

**Fig. 6:**
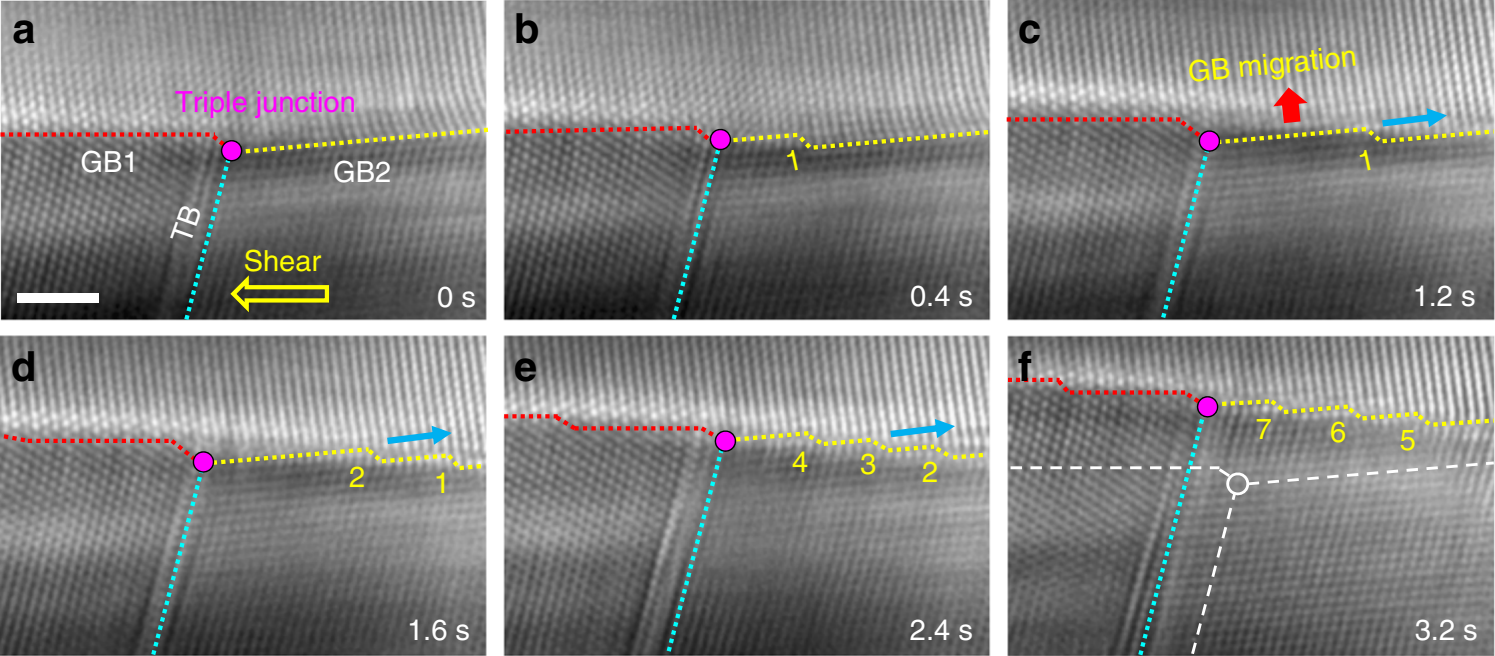
Nucleation of GB disconnections from the triple junction under shear loading. **a** High resolution TEM image showing an Au nanostructure with a triple junction (marked out by the purple dot) connecting two general tilt GBs (denoted as GB1 and GB2, respectively) and a twin boundary (TB). A leftward shear stress was applied to the bottom grains, as shown by the yellow arrow. **b** Nucleation of the first disconnection 1 from the triple junction, which propagated onto the GB2. **c** Upward migration of the GB2 via the lateral motion of disconnection 1. **d** Nucleation of disconnection 2 from the GB-TB triple junction. **e**, **f** Further upward migration of GB2 induced by the successive nucleation and lateral motion of disconnections. The white dashed lines mark out the initial positions of the GBs and the TB; the blue and red arrows indicate the directions of disconnection motion and GB migration, respectively. Scale bar: 2 nm

In real materials, other types of defects (*e.g*. dislocation or stacking fault) may co-exist with the GB disconnection and influence the corresponding deformation behaviour. To further address this question, some stacking faults were artificially introduced into an Au bicrystal with the Σ11(113) GB for additional testing. Interestingly, similar disconnection-dominated GB migration was observed in the presence of pre-existed stacking faults (Fig. 5a–c), indicating that the disconnection motion is an inherent migration mechanism of the Σ11(113) GB. More importantly, such disconnection-mediated GB migration seemed to be common in Au bicrystals with different structures, not just restricted to the specific Σ11(113) GB demonstrated above. For example, Fig. 5d–f show that the disconnection motion also dominated the migration of a *θ* = 57° < 110 > asymmetrical tilt GB, even in the presence of numerous full dislocations near the GB. As shown in Fig. 5d, e, a disconnection (marked out by the aqua circle) moved continuously toward left and passed a pair of GB dislocations without any evident interaction. Upon subsequent loading, the GB migrated upward through the successive nucleation and propagation of GB disconnections, while the pre-existed dislocations remained almost static (Fig. 5f). Figure 6 further presents that such disconnection mechanism also dominated the migration of a tilt GB bound by a triple junction. Given that the disconnection can also contribute to the GB migration in bulk polycrystalline materials, such as the Σ = 51b and 76.4° < 001 > GBs^25,39^, the disconnection-mediated GB plasticity should be a general deformation mode in crystalline materials with different GBs and the atomistic observations presented here demonstrate a straightforward view of these GB processes in experiment.

It is also noticed that the disconnections nucleated predominantly from the free surface in our nanosized bicrystal samples. In bulk polycrystalline materials, the triple junctions may serve as effective nucleation sites upon deformation, especially in the samples with small grain sizes where the dislocation activities were suppressed^24^. Figure 6 presents a direct experimental observation of this mechanism in an Au nanostructure with a triple junction connecting two general tilt GBs and a twin boundary (TB). Upon the leftward shear loading, the first disconnection (denoted as 1) was emitted from the GB-TB triple junction (Fig. 6b), resulting in an upward migration of GB2 via its lateral motion. Further migration of GB2 was mediated by the layer-by-layer nucleated disconnections from the triple junction (Fig. 6d–f), consistent with the mechanism observed in the Σ11(113) coherent GB (Fig. 1). The experimental results presented in Figs. 5–6 suggest that the disconnection-mediated GB deformation should be a prevalent deformation mechanism in bulk polycrystalline and nanocrystalline materials, where the triple junctions can act as effective nucleation sites of GB defects.

## Discussion

Previous studies have reported the disconnection-mediated GB migration in bulk polycrystalline FCC metals with different GBs and in cubic zirconia^23–25,40^. However, the mechanism of disconnection-mediated migration still involved contradictory conclusions, especially about the roles of different types of GB disconnections^7,27,28^. These contradictions may originate from the fact that the frequent composition and decomposition between different types of GB disconnections observed in our experiments were usually neglected in previous theoretical studies due to the unclear disconnection dynamics. With the state-of-art in situ shear experiments, we overcame the technical challenges and clearly demonstrated the dynamic interplay between disconnections at atomic scale. These dynamic processes dominated the migration of different GBs (Figs. 1, 5, and 6). Given that the step configuration constituted the primary processes in different GB migration models^11,18^ and has been widely observed during the shear-coupled migration of different types of GBs in bulk bicrystals or polycrystalline materials (with < 001 > and < 110 > tilt GBs)^12,24,25^ and in current work (Figs. 1, 5 and 6), the dynamic processes of GB disconnections and the resultant GB migration reported here should have general implications to the understanding of GB-dominated plasticity in both bulk and nanosized crystalline materials. It is noticed that the pre-existed defects (*e.g*. dislocation or stacking fault) seemed to have only limited influence on the disconnection-mediated GB migration (Fig. 5). Particularly, neither interplay between disconnection and pre-existed defects nor change of disconnection configuration occurred when a disconnection moved and passed the pre-existed GB dislocations or lattice dislocations (Fig. 5d–f). This unique behaviour may originate from the fact that the small disconnections (with a height of one or two atomic layers) only bear a low lattice resistance for their motion^41^, favouring the disconnection-mediated deformation. However, it needs to be noted that several factors (e.g. GB structure, loading condition and temperature) may affect the disconnection configuration, which can influence the dynamic behaviour of disconnections and their interaction/competition with other defects. For example, high temperature heating can introduce other type of disconnection (*e.g*. four-layer) in Σ11(113) GB^23^, and different disconnection configurations were observed in the Σ41 < 001 > {540} GB due to the variation of GB core structures^24^. Additional experimental and simulation studies are required in order to obtain a systematic understanding on the nucleation and dynamic behaviour of GB disconnections under different conditions, as well as their interaction/competition with other defects.

Our experiments also demonstrated that the disconnection-mediated GB migration was highly reversible in loading cycles, like the twinning-induced pseudoelasticity in metallic nanowires^37,38^, both of which showed a layer-by-layer growth and reversible deformation behaviour. This unique behaviour could benefit the cyclic deformation of nanostructured materials, providing a model system for the future theoretical study of load-bearing nanostructures. It is also noticed that although the disconnection activities can contribute to the migration of different types of GBs (e.g. < 001 > and < 110 > tilt GBs), the mechanism presented in this paper cannot represent the migration of all types of GBs, since a number of GBs in real crystalline materials cannot be characterised merely by the GB disconnections. Nonetheless, the state-of-art in situ nanofabrication provides an effective way to produce metallic nanostructures with different types of GBs (see Supplementary Fig. 11 for additional examples), which opens new opportunities to systematically investigate various modes of GB-dominated plasticity in near future.

In conclusion, atomistic migration mechanism of Σ11(113) coherent GB in Au bicrystals at room temperature was revealed using a state-of-art in situ shear testing technique inside TEM combined with MD simulations. Both single-layer and double-layer disconnections can contribute to the GB migration through frequently-occurred dynamic composition and decomposition. Besides, the shear-induced migration of Σ11(113) GB was fully reversible in loading cycles. Given that this disconnection-mediated GB deformation can occur in different GBs and the triple junctions usually serve as effective nucleation sites of GB defects, the disconnection-mediated GB deformation should be a general deformation phenomenon in polycrystalline and nanocrystalline materials, providing new insight into the GB-dominated plasticity.

## Methods

### In situ TEM shear deformation

In situ nanofabrication and shear testing of Au bicrystals with Σ11(113) coherent GBs were carried out inside a FEI Titan Cs-corrected TEM, using a TEM electrical holder from Beijing PicoFemto Co. Before experiment, Au rod with the purity of 99.99 wt.% and the diameter of 0.25 mm was ordered from Alfa Aesar Inc. The experimental setup is illustrated in Supplementary Fig. 11. In a typical experiment, a bulk Au rod was fractured by a ProsKit wire cutter to obtain a clean fracture surface with numerous nanoscale tips, which was then loaded onto the static side of the TEM holder; meanwhile, another fractured Au rod was loaded onto the probe side of the TEM holder, which was controlled by the piezo-manipulator to handle the sample. Then, the Au rod on the probe side was moved to contact with the rod on the static side of the holder. To make an Au bicrystal with Σ11(113) coherent GB, two nanoscale tips on the Au rods of static and probe sides were selected, such that they were oriented in the < 110 > zone axis; subsequently, the two nanoscale tips were welded together in situ inside the TEM by properly choosing a welding site. Before welding, a voltage potential of −1.5 V was pre-applied on the nanoscale tip at the probe side to enhance the weldability. At the moment of contact, the pre-applied potential could melt these two nanoscale tips together and an Au bicrystal was thus formed due to the orientation differences between the nanoscale tips. Similarly, Au bicrystals with other types of GBs were in situ fabricated by changing the orientation differences between the two nanoscale tips on both sides. For the fabrication of Au nanostructure with a triple junction, an Au tip with a TB on the static side was selected and then welded with the single crystal Au tip on the probe side. During in situ shear testing experiment, the Au rod on the probe side was controlled to move leftward/rightward slowly to carry out the shear deformation at a constant rate of ~0.005 nm s^−1^, giving an estimated strain rate of 10^−3^ s^−1^. Throughout the shear loading process, the zone axes of both upper and bottom grains were observed to keep nearly constant, despite the unavoidable mechanical vibrations (Supplementary Fig. 12). In all experiments, the TEM was operated at 300 kV and weak beam conditions were used to minimise the potential beam effects on deformation. Besides, all in situ experiments were recorded in real time by a charge-coupled device (CCD) camera at a rate of ~0.3 s per frame. It needs to be noted that the in situ nanofabrication method reported here is also applicable to fabricate different types of GBs (*e.g*. high angle GB, low angle GB and mixed GB) in FCC metals, which opens new opportunities to systematically study the GB-dominated deformation at atomic scale.

### Molecular dynamic simulations

MD simulations were carried out using an Au bicrystal model with a total ~67,000 atoms using Large-scale Atomic/Molecular Massively Parallel Simulator (LAMMPS)^42^ and embedded atom method (EAM) potentials for Au^43^. A bicrystal model was created by constructing two separate crystal lattices with the crystallographic misorientation corresponding to Σ11 symmetrical GB (*θ* = 51°) and joining them together along the < 113 > direction. The equilibrium GB structure was prepared using an energy minimisation procedure and a number of initial positions of the two grains were tested to determine the most energetically favoured GB structure. Periodic boundary conditions were applied along [110] direction while the other directions were set free. In the shear deformation, a constant shear velocity v = 1 m s^−1^ parallel to the boundary plane was applied on the fixed area of the bottom grain along the < 332 > direction at 300 K while fixing a few atom layers at the top of the upper grain. Figure 2i–n, Supplementary Fig. 8 and Supplementary Fig. 10 show the simulated results of the shear deformation of the Au bicrystal. To determine the nucleation energy barriers for the single-layer and double-layer disconnections, as well as their dynamic composition/decomposition processes, disconnections were created in the originally defect-free model by translating part of the bottom grain along the DSC Burgers vector^27^, and then the MD simulations were performed at 0 K via the energy minimisation. Supplementary Table 1 summarised the energy barriers for different GB disconnections, and the dynamic composition/decomposition of the single-layer and double-layer disconnections were presented in Fig. 3g–j and Supplementary Fig. 7. The visualisation tool Ovito^44^ was used to illustrate the bicrystal model and common neighbour analysis modification was applied to clearly identify the GB structure and its evolution during the simulations. Three categories of atoms were identified in our system, including atoms of FCC, hexagonal close-packed and other structure orders. The Burgers vector can be determined by drawing a Burgers circuit around the disconnection core.

## Supplementary information

Supplementary Information

Description of Additional Supplementary Files

Supplementary Movie 1

Supplementary Movie 2

## Data Availability

The data that support the findings of this study are available from the corresponding authors upon request.
