# Supplementary material for: In situ atomistic observation of disconnection-mediated grain boundary migration
Source: Nat Commun. 2019 Jan 11;10:156. doi: 10.1038/s41467-018-08031-x (PMC6329749; doi:10.1038/s41467-018-08031-x)
Supplement: Supplementary file 1 — Supplementary Information [file 41467_2018_8031_MOESM1_ESM.pdf]

Supplementary Information

In situ atomistic observation of disconnection-mediated grain boundary migration

Zhu et al.

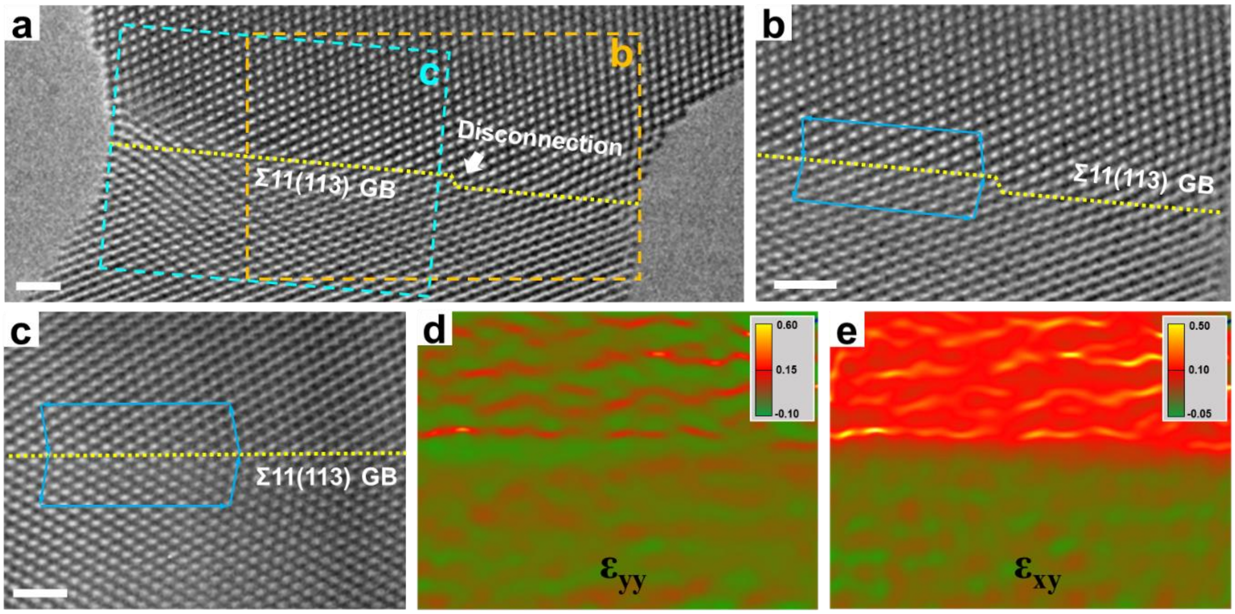

Supplementary Figure 1. Atomistic lattice structure of the Au bicrystal presented in Figure 1. (a) High resolution transmission electron microscopy (TEM) image of the Au bicrystal, without observable lattice and grain boundary (GB) defect other than the disconnection. (b-c) Perfect Burgers circuits (blue) around GB indicate that no dislocation exists on the GB near (b) or away from (c) the disconnection, which are marked out by the orange and aqua rectangles in (a), respectively. (d-e) Geometric phase analysis (GPA) of the lattice in (c), confirming the absence of both GB dislocation and lattice dislocation in the as-fabricated Au bicrystal. Scale bars: 1 nm.

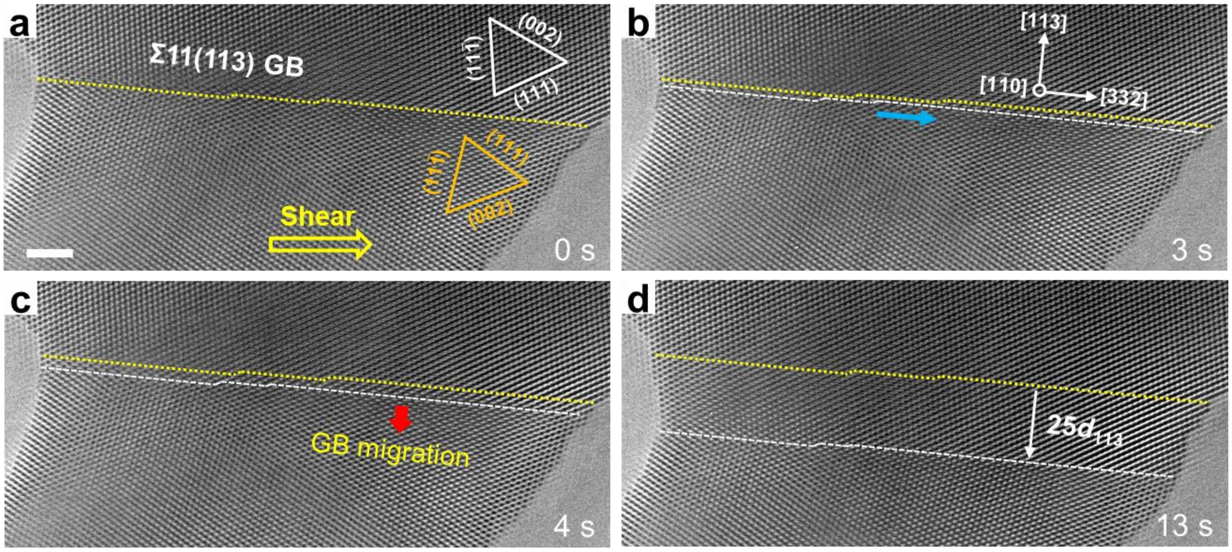

Supplementary Figure 2. Additional example showing the disconnection-mediated migration of a Σ11(113) GB. A shear stress was applied to the bottom grain toward right, inducing the lateral motion of GB disconnections and thereby downward migration of the GB. The migration directions of GB disconnection and Σ11(113) GB are marked out by the blue and red arrows, respectively. The yellow dotted lines and white dashed lines indicate the initial and current positions of the GB, respectively. Scale bar: 2 nm.

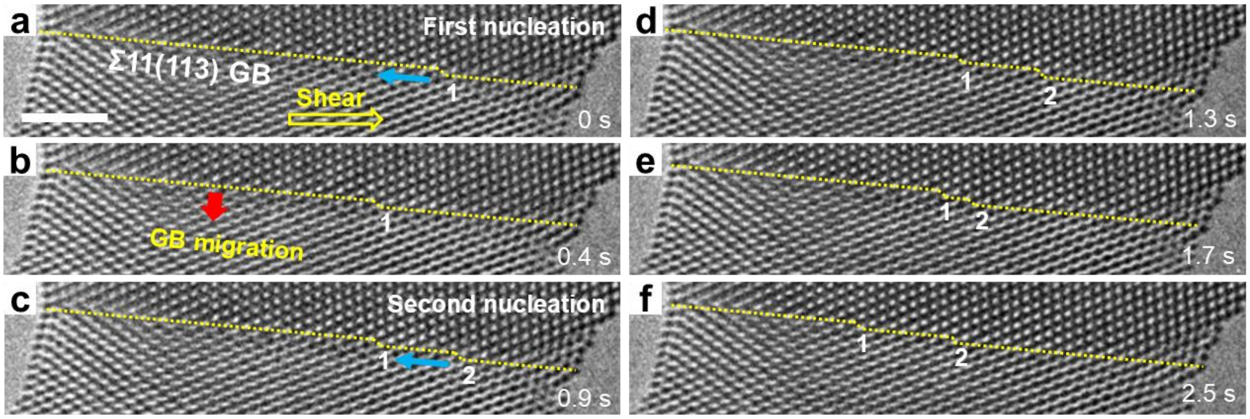

Supplementary Figure 3. Successive nucleation and lateral motion of disconnections coupled to the migration of a Σ11(113) coherent GB. (a-b) Nucleation and lateral motion of the first disconnection on the (113) GB plane, denoted as 1. The directions of shear stress, disconnection motion and GB migration are indicated by the yellow, blue and red arrows, respectively. (c) Nucleation of the second disconnection (denoted as 2) on the neighbouring (113) plane. (d-f) Co-existence and lateral motion of these two single-layer disconnections. The lateral motion rates of disconnections 1 and 2 are measured to be ~1.98 nm s^{-1} and ~1.91 nm s^{-1}, respectively. Scale bar: 2 nm.

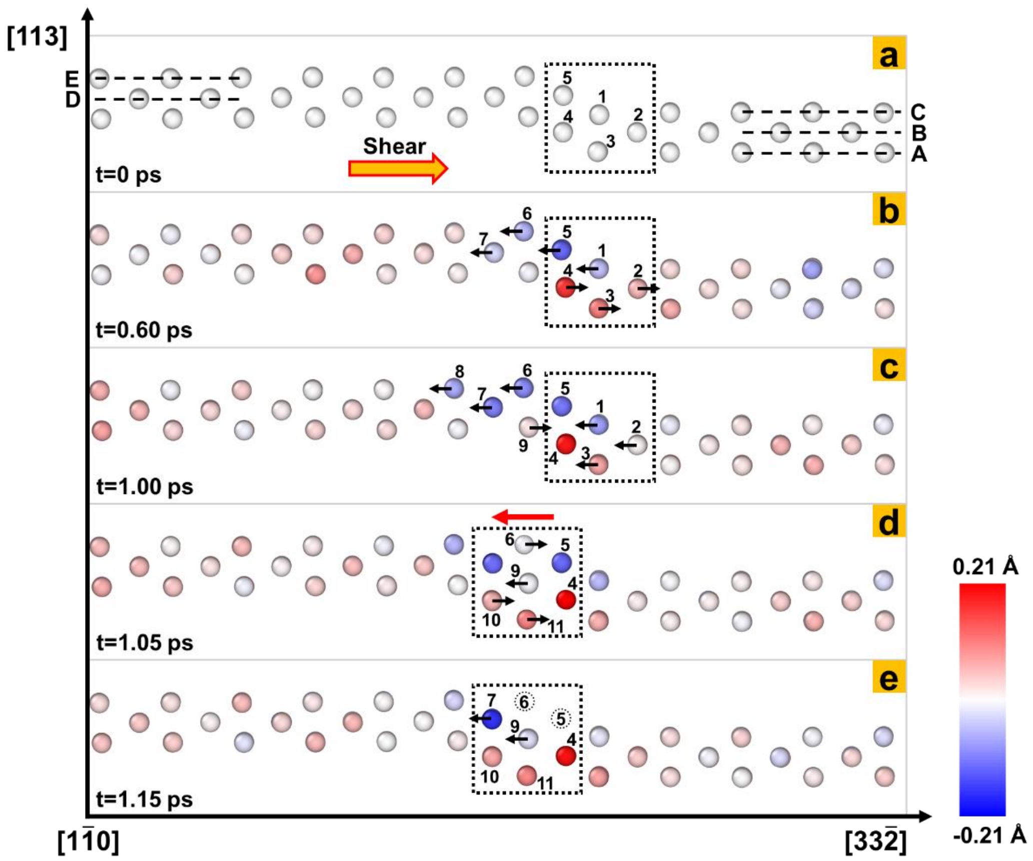

Supplementary Figure 4. Atomistic dynamics of a double-layer disconnection under shear loading by molecular dynamics (MD) simulation. (a) Structure of the Σ11(113) coherent GB with a double-layer disconnection. Five layers of atoms (denoted as A-E) were quantitatively analysed, with layers B and D corresponding to the GB planes on either side of the disconnection core (marked out by the square box). The atoms near the Σ11(113) coherent GB were coloured based on their local displacement along $[33\bar{2}]$ direction, as indicated in the colour map. (b) The pure shear loading along $[33\bar{2}]$ caused a leftward motion of atom columns 1 (disconnection core) and 5-7, as well as a rightward motion of atom columns 2-4, as indicated by the black arrows. (c) The lateral motion of disconnection core was incubated by the leftward displacement of atom columns 6 and 7, which further triggered the motion of atom column 8. Meanwhile, the motion of atom columns 2 and 3 was reversed toward left. (d) Atom column 6 reversed its displacement towards the equilibrium position while atom columns 10 and 11 started to deviate from the face-centred cubic (FCC) lattice, constituting the new disconnection core. As a result, one-step lateral motion of the GB disconnection core was realized (indicated by the red arrow), with the atom column 9 becoming the new core centre. (e) Relaxation of the disconnection core caused the atom columns 5 and 6 returning to the FCC lattice (indicated by the dotted circles), while the atom columns 7 and 9 continued to move leftward, contributing to the next step of disconnection motion. The atomistic displacement vector analysis clearly demonstrates that the lateral motion of disconnection was merely controlled by the localized fluctuations of atoms in the disconnection core around their equilibrium positions, without the involvement of any conventional lattice defect.

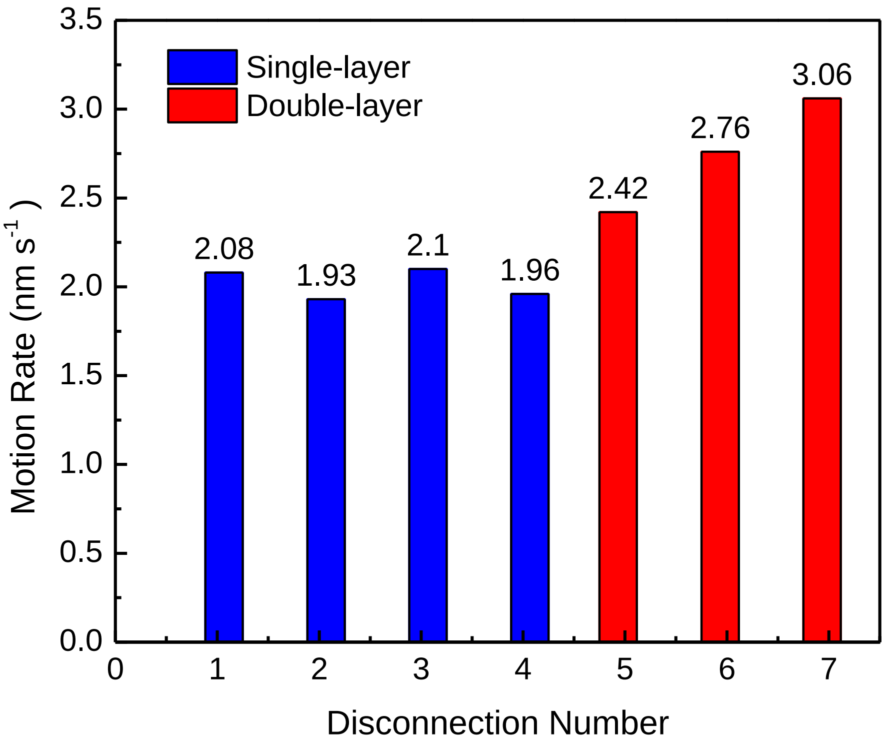

Supplementary Figure 5. Statistical measurement of disconnection motion rates during the migration of a Σ11(113) GB. Clearly, the motion rates of GB disconnections show some variations, and the motion rates of single-layer disconnections are typically lower than those of double-layer disconnections. The average motion rates of single-layer and double-layer disconnections are ~2.02 nm s^{-1} and ~2.75 nm s^{-1}, respectively.

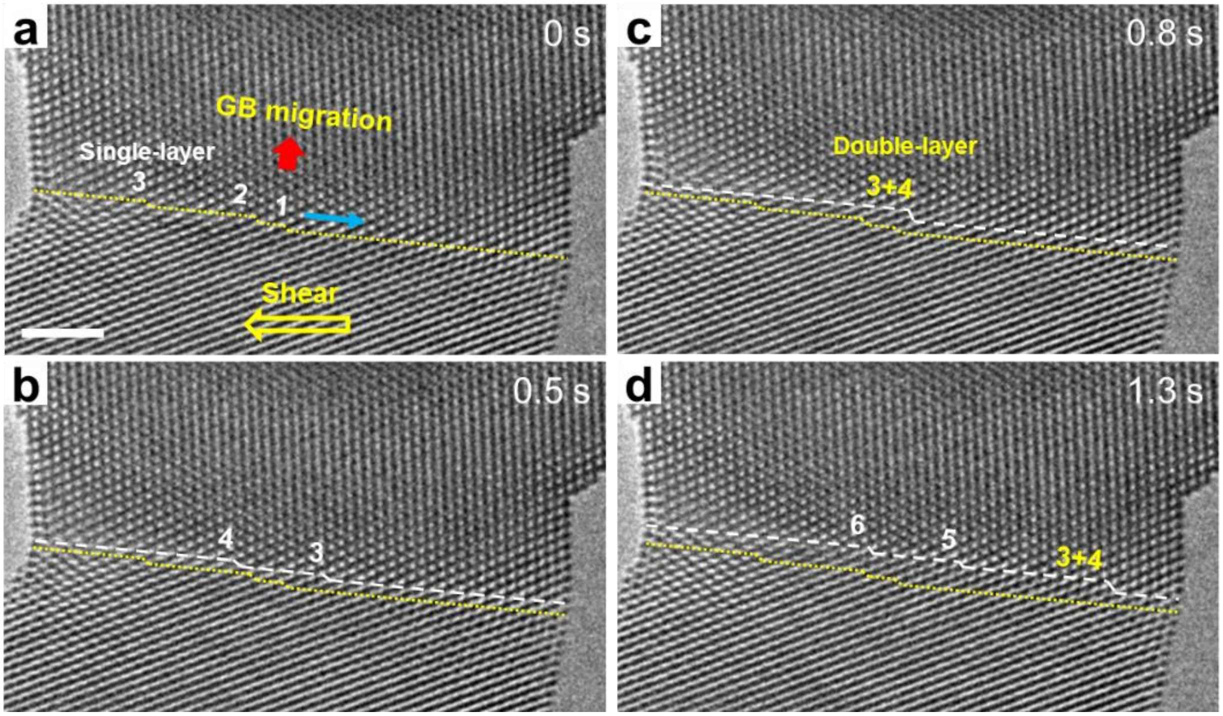

Supplementary Figure 6. Dynamic composition of the single-layer disconnections during the *in situ* shear deformation. (a) A Σ11(113) coherent GB with three pre-existed single-layer disconnections (denoted as 1, 2 and 3, respectively). (b) Nucleation of another single-layer disconnection (denoted as 4). During this process, the disconnections 1 and 2 moved and annihilated to the free surface quickly, resulting in the upward migration of the GB. (c) Dynamic composition of two single-layer disconnections (3 and 4) into a double-layer one (3+4). (d) Lateral motion of the newly-formed double-layer disconnection (3+4), which was followed by the emission of two single-layer disconnections (5 and 6) from the free surface. The yellow dotted lines and white dashed lines represent the initial and current positions of the GB, respectively; the directions of shear stress, disconnection motion and GB migration are indicated by the yellow, blue and red arrows, respectively. Scale bar: 2 nm.

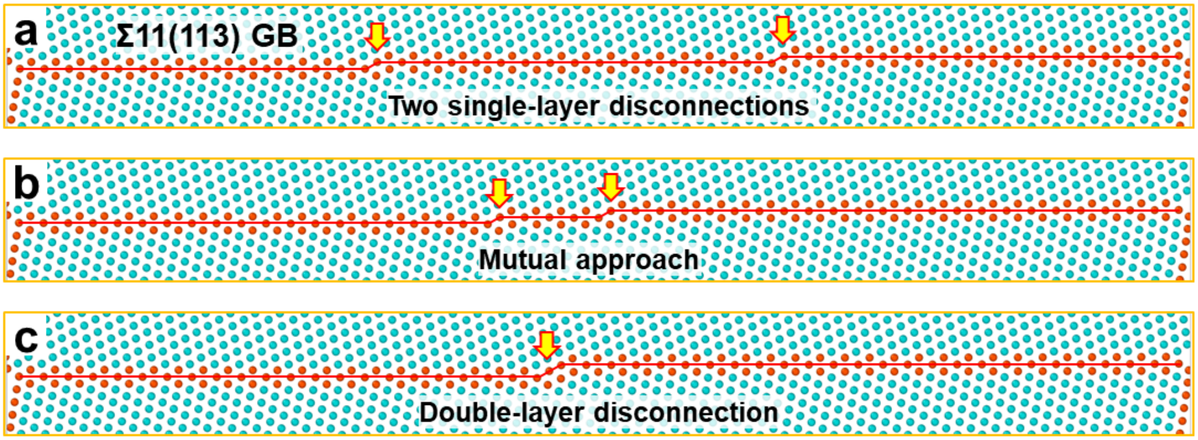

Supplementary Figure 7. MD simulation of the energetically favourable composition of single-layer disconnections. The MD simulation was performed at 0 K by the energy minimization to determine the energy difference between the two single-layer disconnections and the double-layer disconnection. (a) A Σ11(113) coherent GB with two pre-existed single-layer disconnections (pointed out by the yellow arrows) in a defect free bicrystal model. (b-c) Spontaneous composition of the two single-layer disconnections into a double-layer disconnection, induced by the energy minimization of the system.

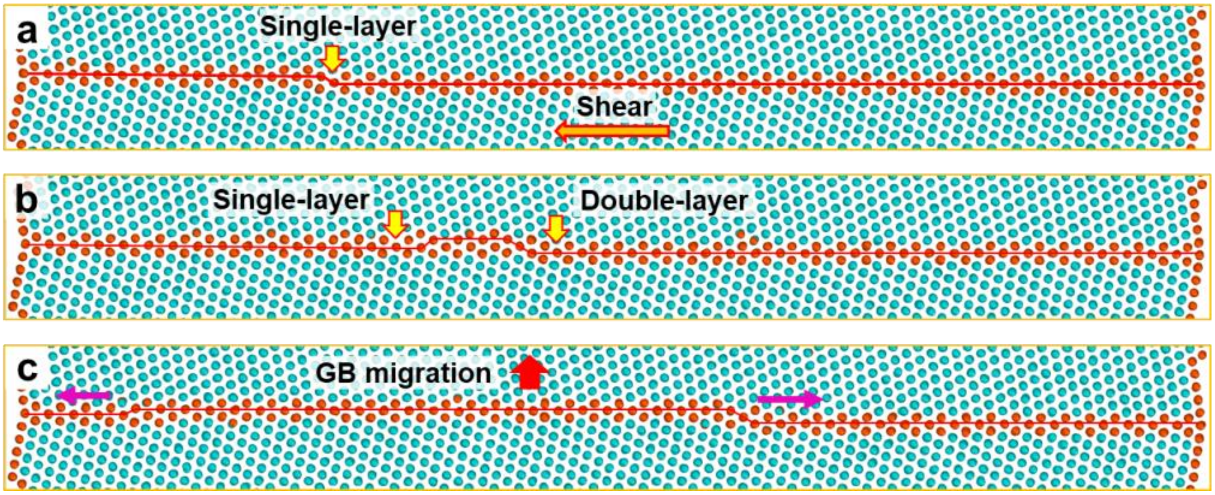

Supplementary Figure 8. MD simulation of dynamic dissociation of a single-layer disconnection. The MD simulation was performed at 300 K with a shear stress (indicated by the orange arrow) applied to the lower grain with a constant velocity of 1 m s^{-1}. (a) A single-layer disconnection (pointed out by the yellow arrow) was emitted from the free surface under shear loading. (b) Dissociation of the single-layer disconnection into a kinked disconnection dipole, composed of a single-layer (left) and a double-layer (right) disconnections. (c) Further shear loading caused the lateral motions of the two components of the disconnection dipole, which moved oppositely and contributed to the GB migration. The directions of disconnection motion and GB migration are shown by the purple and red arrows, respectively.

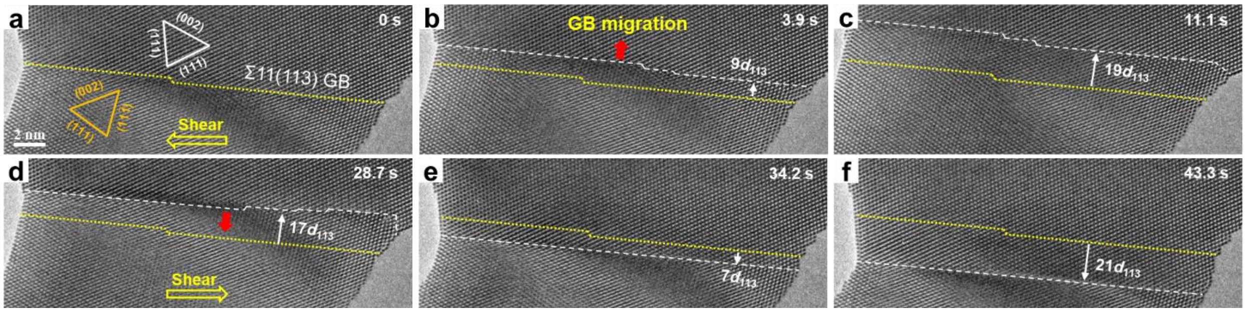

Supplementary Figure 9. **Additional example showing the reversible migration of a Σ11(113) coherent GB in shear loading cycles.** (a-c) Upward migration of the (113) GB under the leftward shear loading. (d-e) Reverse (downward) migration of the (113) GB under the rightward shear loading. The yellow dotted lines and white dashed lines represent the initial and current positions of the GB, respectively. The migration distances are shown in each image accordingly. Scale bar: 2 nm.

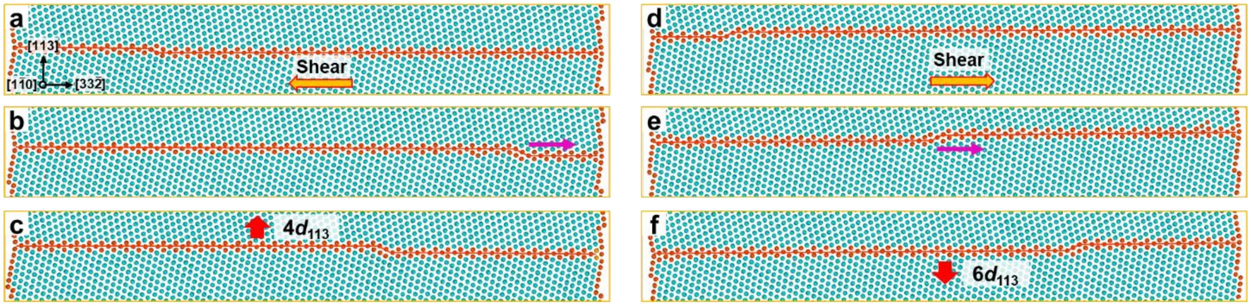

Supplementary Figure 10. **MD simulation of reversible migration of a Σ11(113) GB in shear loading cycles**. The MD simulation was performed at 300K with a shear loading applied to the bottom grain at a velocity of 1 m s^{-1}. (a-c) Upward migration of the (113) GB under shear stress along $[\bar{3}\bar{3}2]$. (d-f) Reversible (downward) migration of the (113) GB under shear stress along $[33\bar{2}]$.The upward and downward migration distances of the GB are indicated in (c) and (f), respectively. The directions of shear stress, disconnection motion and GB migration are marked out by the orange, purple and red arrows, respectively.

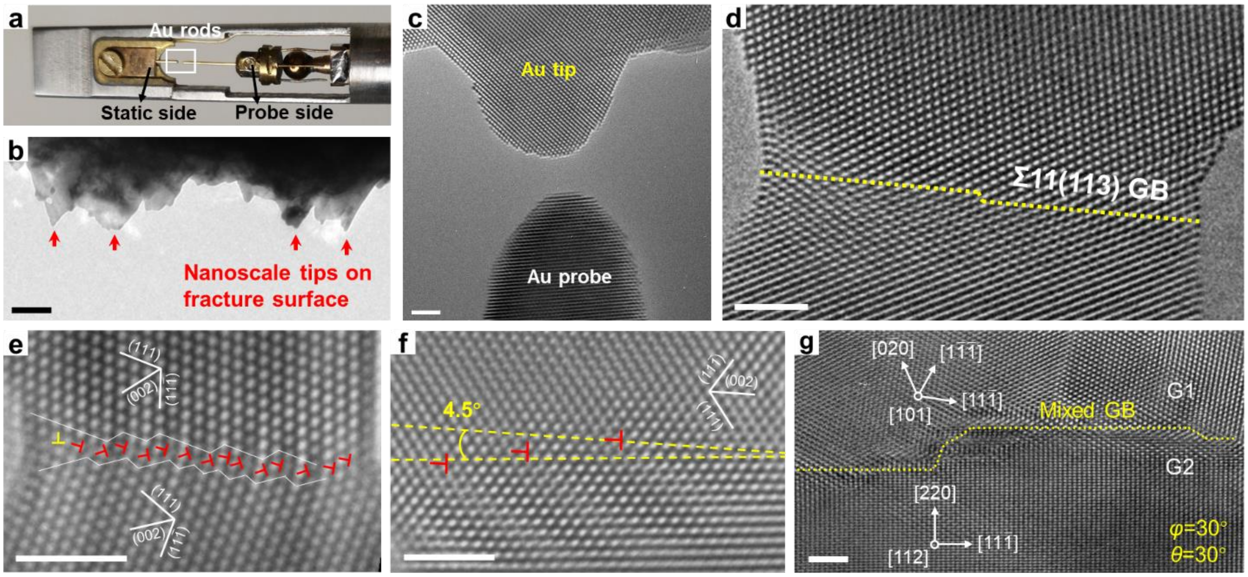

Supplementary Figure 11. Experimental setup of the *in situ* nanofabrication and the as-fabricated Au bicrystals with different GBs. (a) The TEM-STM platform used in current experiments. (b) Nanosized tips on the fracture surfaces of an Au rod. (c) A nanoscale tip on the probe side was driven to contact with an Au tip on the static side while a potential of -1.5 V was pre-applied on the probe side. (d) Au bicrystal with the Σ11(113) GB fabricated upon contact. (e-g) Different GBs fabricated by nano-welding inside TEM. (e) A θ=20° <110> high angle GB. (f) A θ=4.5° <110> low angle GB. (g) A mixed GB with a twist angle (φ) of 30° and a tilt angle (θ) of 30°. Scale bars: (b) 100 nm, (c-g) 2 nm. Given that the *in situ* nanofabrication method reported here is also applicable to fabricate different types of GBs (see (e-g) of this Figure, and Fig. 1, Fig. 5-6 in the main text) in FCC metals, this method opens a new opportunity to systematically study the GB-dominated deformation at atomic scale.

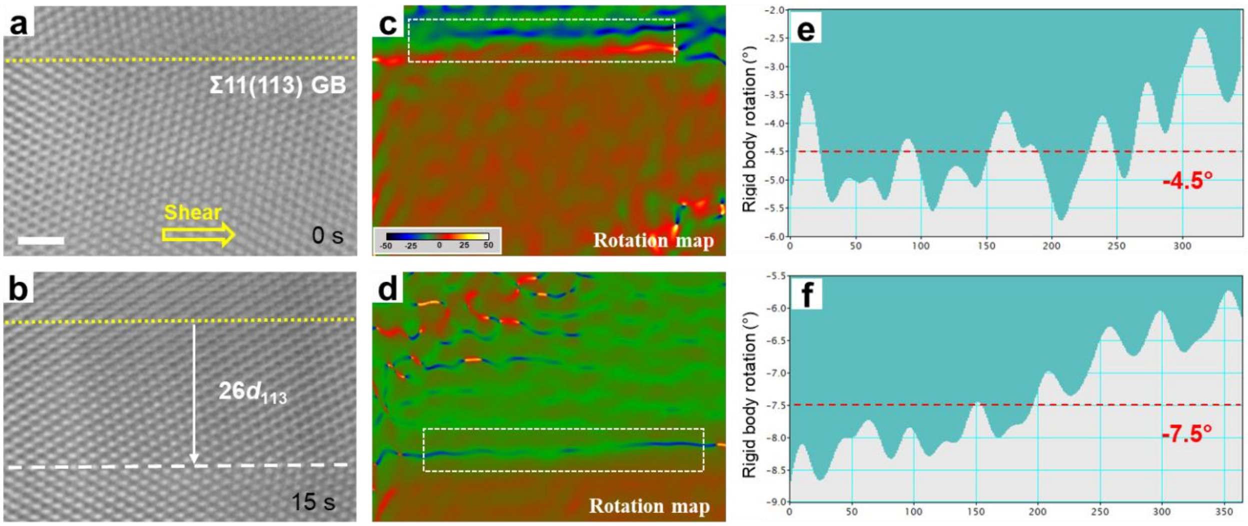

Supplementary Figure 12. GPA of the relative rotation between the two grains of an Au bicrystal during deformation. (a-b) Downward migration of a Σ11(113) GB under the rightward shear stress (shown by the yellow arrow). The yellow dotted lines and white dashed line represent the initial and final positions of the GB, respectively. (c-d) Corresponding lattice rotation maps of (a-b) obtained from GPA. (e-f) Line profiles along the (113) GB showing a slight change of the relative rigid body rotation between the upper (static side) and bottom (probe side) grains before and after the GB migration, from the initial -4.5° to the -7.5° under shear loading. The line profiles in (e) and (f) are obtained by averaging the lattice rotation over the width of the box in (c) and (d), respectively. Scale bar: 2 nm.

Supplementary Table 1. Surface nucleation energies of different disconnection configurations

**Disconnection**	**Burgers Vector**	**Nucleation Energy**
Single-layer	$\mathbf{b}_1$ or $\mathbf{b}_2$ = (1/22) <471>	1.61 eV
Double-layer	$\mathbf{b}_3$ = (1/22) <332>	3.17 eV
Two single-layer (separated)	$\mathbf{b}_1$ and $\mathbf{b}_2$	4.44 eV
Two single-layer (composited)	($\mathbf{b}_1$ + $\mathbf{b}_2$)	2.83 eV

***** The MD simulations were performed at 0 K by energy minimization.
