# Supplementary material for: In situ atomistic observation of disconnection-mediated grain boundary migration
Source: Nat Commun. 2019 Jan 11;10:156. doi: 10.1038/s41467-018-08031-x (PMC6329749; doi:10.1038/s41467-018-08031-x)
Supplement: Supplementary file 2 — Description of Additional Supplementary Files [file 41467_2018_8031_MOESM2_ESM.pdf]

Description of Additional Supplementary Files

File Name: Supplementary Movie 1
Description: Shear-induced deformation of an Au bicrystal nanojunction with a Σ11(113) coherent GB. The migration of the GB was mediated by the lateral motion of GB disconnections. The movie was speeded up by 5 times.

File Name: Supplementary Movie 2
Description: Fully reversible migration of a Σ11(113) coherent GB in an Au bicrystal nanojunction during the shear loading cycles. The movie was speeded up by 10 times.
